# The complex relationship between obesity and neurodegenerative diseases: an updated review

**DOI:** 10.3389/fncel.2023.1294420

**Published:** 2023-11-09

**Authors:** Alexandre Neto, Adelaide Fernandes, Andreia Barateiro

**Affiliations:** ^1^Central Nervous System, Blood and Peripheral Inflammation, Research Institute for Medicines (iMed.ULisboa), Faculdade de Farmácia, Universidade de Lisboa, Lisbon, Portugal; ^2^Department of Pharmaceutical Sciences and Medicines, Faculdade de Farmácia, Universidade de Lisboa, Lisbon, Portugal

**Keywords:** adipokines, Alzheimer’s disease, cognitive impairment, inflammation, multiple sclerosis, neurodegenerative diseases, obesity, Parkinson’s disease

## Abstract

Obesity is a global epidemic, affecting roughly 30% of the world’s population and predicted to rise. This disease results from genetic, behavioral, societal, and environmental factors, leading to excessive fat accumulation, due to insufficient energy expenditure. The adipose tissue, once seen as a simple storage depot, is now recognized as a complex organ with various functions, including hormone regulation and modulation of metabolism, inflammation, and homeostasis. Obesity is associated with a low-grade inflammatory state and has been linked to neurodegenerative diseases like multiple sclerosis (MS), Alzheimer’s (AD), and Parkinson’s (PD). Mechanistically, reduced adipose expandability leads to hypertrophic adipocytes, triggering inflammation, insulin and leptin resistance, blood-brain barrier disruption, altered brain metabolism, neuronal inflammation, brain atrophy, and cognitive decline. Obesity impacts neurodegenerative disorders through shared underlying mechanisms, underscoring its potential as a modifiable risk factor for these diseases. Nevertheless, further research is needed to fully grasp the intricate connections between obesity and neurodegeneration. Collaborative efforts in this field hold promise for innovative strategies to address this complex relationship and develop effective prevention and treatment methods, which also includes specific diets and physical activities, ultimately improving quality of life and health.

## 1. Obesity

In recent years, obesity, a metabolic disorder, has changed from a mere aesthetic problem to a serious health problem worldwide. Nowadays, obesity is declared by medical authorities as a global 21st-century epidemic and represents one of the leading public health problems throughout the world since its prevalence is constantly increasing in developed and developing countries, being a significant risk factor for several diseases. The body mass index (BMI) is the most widely used method to classify an individual’s body type concerning their respective weight and height. A BMI of 18.5–24.9 kg/m^2^ is considered a normal healthy weight, between 25.0 and 29.9 is considered overweight, 30.0–39.9 means obese, and from 40.0 upwards means severely obese (Lloret et al., 2019). Worldwide, about 2.1 billion people (30% of the global population) are overweight, of which over 650 million are obese, but more worrisome is the rapidly increasing prevalence of this disease over the last 30 years, since it nearly triplicated between 1975 and 2016 (World Health Organization, 2018; Chooi et al., 2019)^1^. If the increased incidence continues at the current pace, it is predicted that almost half of the world’s adult population will be obese or overweight by 2030 (Tremmel et al., 2017). The prevalence of obesity has also increased in children and adolescents in impressive proportions, and more than 40 million children (<5 years) are considered obese in the world (Llewellyn et al., 2016; World Health Organization, 2018)^1^. Some studies have also shown significant differences in the prevalence of obesity due to gender, which is more commonly observed in women (Chooi et al., 2019).

The World Health Organization has defined obesity as a state in which the individual contains excessive or abnormal body fat accumulation due to a positive energy balance characterized by excessive energy intake and insufficient expenditure. Obesity is a multifactorial pathology caused by diverse genetic, behavioral, societal, and environmental factors. Several studies have shown that obesity is highly heritable, with genetics predisposing certain ethnicities and individuals to develop obesity due to numerous genes related to adiposity and weight gain (Grarup et al., 2014). However, several factors, such as overconsumption of energy-density foods, carbohydrates, high-sugar foods, reduced physical activity, and a sedentary lifestyle, are mainly responsible for gaining weight. Moreover, endocrine factors can contribute to obesity, like hyperinsulinism, hypercortisolism, ovarian dysfunction, and hypothyroidism. Finally, weight gain can also be caused by taking medications, such as steroid hormones and psychoactive drugs (Hruby and Hu, 2015).

As mentioned before, the etiology of obesity encompasses the positive energy imbalance when the daily ingested calories surpass the caloric burning, leading ultimately to an excess of white adipose tissue (WAT). This tissue stores energy as triglycerides and increases its size due to the expansion of the adipocytes both through size expansion (hypertrophy) and number (hyperplasia) (Chouchani and Kajimura, 2019). Although initially the adipose tissue was considered an inert fat storage depot, this view was completely changed with several studies demonstrating that it is a complex organ with endocrine functions that controls and modulates energy storage and accumulation, having the ability to synthesize and secrete hormones and adipokines that have a large array of biological effects on metabolism, homeostasis, and inflammation (Sethi and Vidal-Puig, 2007; Poulos et al., 2010). The adipose tissue function and secretome are tightly controlled by complex homeostatic mechanisms and local cell-cell interactions, which can become dysregulated in obesity. In this context, the chronic unnecessary expansion of the WAT is harmful, since it ultimately leads to obesity in part due to a chronic inflammatory status, which impairs both energy balance and immune function regulation (Speakman, 2010). In these situations, local demand for blood exceeds supply and a fraction of adipocytes become stressed or die due to hypoxia, leading to the liberation of damage-associated molecular patterns. Consequently, these damage-associated molecular patterns attract immune cells such as macrophages and leukocytes that exacerbate the pro-inflammatory milieu with the release of pro-inflammatory factors, such as tumor necrosis factor-α (TNF-α), interferon (IFN)-γ, interleukin (IL)-1β, IL-18, IL-6, and C-reactive protein, leading to a moderate chronic inflammation within the adipose tissue (Lumeng et al., 2007; Reilly and Saltiel, 2017).

Deeply involved in obesity is the hormone leptin. This hormone is responsible for food intake regulation, as well as energy expenditure, mediated through the respective receptors present in the hypothalamus (Mantzoros et al., 2011). Moreover, in obesity, leptin levels are generally increased and support pro-inflammatory immune responses against the central nervous system (CNS) (Sanna et al., 2003; Matarese et al., 2005). In addition, it is known that the inflammatory response is activated early in the adipose expansion and during chronic obesity, permanently diverting the immune system to a pro-inflammatory phenotype, leading to a reciprocal influence of obesity and inflammation (Saltiel and Olefsky, 2017).

The low-grade inflammation that is tightly related to the physiopathology of adipose tissue is a common feature of all obesity-related diseases (Lionetti et al., 2009) like metabolic syndrome, type 2 diabetes mellitus, insulin resistance, fatty liver disease, arteriosclerosis, several cancers; as well as several mental disorders, dementia, and neurodegenerative diseases (Ozcan et al., 2004; Profenno et al., 2010; Anstey et al., 2011; Friedemann et al., 2012; De Pergola and Silvestris, 2013; Mandviwala et al., 2016; Tronieri et al., 2017; Arshad et al., 2018).

So, obesity, the modifiable risk factor for several diseases, such as neurodegenerative diseases, is receiving growing attention due to the relatively simple and low-cost interventions that could be employed to tackle further comorbidities. In this context, is fundamental to understand the mechanisms by which obesity contributes to the onset and development of neurodegenerative diseases.

## 2. Obesity-related mechanisms and neurodegenerative diseases

Obesity affects various organ physiological functions, contributing to overall health deterioration. Within the brain, obesity could lead to a broad spectrum of homeostatic disruptions such as higher incidence of oxidative stress, inflammation, protein aggregation, mitochondrial dysfunction, altered hormone levels, insulin resistance, and blood-brain barrier (BBB) compromise (de Bem et al., 2021). All these changes could impair synaptic plasticity and neurogenesis, along with neuronal death, ultimately leading to cognitive function failure (O’Brien et al., 2017). Remarkably, diet-induced obesity (DIO) during early life initiates long-lasting effects through modifications within the innate immune system, persisting beyond the resolution of metabolic problems. Through Toll-like receptor 4, stearic acid modifies chromatin arrangement, heightening availability at binding sites for activator protein-1. This prompts myeloid cells to transition from oxidative phosphorylation to glycolysis, triggering the synthesis of proinflammatory cytokines (Hata et al., 2023).

The abovementioned disruptions, along with obesity’s characterization by a low-grade inflammatory status, have established a link between obesity and neurodegenerative diseases like multiple sclerosis (MS), Alzheimer’s disease (AD), and Parkinson’s disease (PD). This connection provides a significant basis for further exploration into the relationships between these conditions.

### 2.1. Low-grade chronic inflammation

When the adipose expandability is low, the adipose depots are characterized by increased hypertrophic adipocytes, leading to endoplasmic reticulum stress, ultimately activating inflammatory and apoptotic pathways, as well as insulin resistance (Sethi and Vidal-Puig, 2007). Chronic low-grade inflammation, which is distinctive and initially characterized by altered cytokine and adipokine profiles from impaired adipose tissue, stimulates macrophage and lymphocyte recruitment. Moreover, insulin-resistant adipocytes (more lipolytic and less liposynthetic) induce an increase in circulating free fatty acids, which in turn may activate Toll-like receptor 4 in B cells, inducing nuclear factor k-light-chain-enhancer of activated B cells (NF-kB) translocation to the nucleus. Therefore, the subsequent synthesis of pro-inflammatory cytokines such as TNF-α and IL-6 (Rull et al., 2010) contrasts with the down expression of anti-inflammatory molecules as the case of adiponectin (Gariballa et al., 2019). This complex signaling amplifies insulin resistance, lipolysis, and inflammation in the whole adipose tissue. Inflammation is also implicated in the development of diabetes mellitus and insulin resistance, affecting insulin and glucose transport across the BBB (Banks et al., 2012).

Leptin and adiponectin, hormones produced primarily by adipocytes, are involved not only in glucose and lipid metabolism, controlling energy homeostasis, but also in the modulation of inflammation, appearing to play a role in the relationship between obesity and neuronal/glial function (Minokoshi et al., 2002; Yamauchi et al., 2002). With the accumulation of fat mass, leptin levels increase while adiponectin decreases. This unbalanced ratio leads to leptin resistance, and decreased lipid oxidation in non-adipose tissues, ultimately giving rise to lipid accumulation, lipotoxicity, and insulin resistance (Lionetti et al., 2009). Coupled with insulin resistance and hyperleptinemia is the decreased activation of AMP-activated protein kinase (AMPK), a key regulator of cellular metabolism and whole-body energy balance, which has several key functions, such as the regulation of cellular uptake of glucose and free fatty acids, cell cycling, mRNA stability, and apoptosis (Zhou et al., 2017; Gu et al., 2019). In a broader view, it is also capable of controlling appetite, insulin sensitivity and the modulation of adipokines/cytokines (Carling et al., 2011; Hardie, 2011). Moreover, more recent studies showed AMPK ability to regulate lipid metabolism through the enhancement of oxidation and autophagy and, on the other hand, diminishing cholesterol and fatty acid (FA) production (Gu et al., 2018; Herzig and Shaw, 2018). High-fat diet (HFD) fed mice studies have shown a decrease in AMPK activation in WAT, heart, and liver, which may be linked with mitochondrial malfunction, lower FA oxidation, activation of NF-κB signaling, and therefore low-grade metabolic inflammation, oxidative stress and insulin resistance (Crispino et al., 2020). Interestingly, this decrease in AMPK activity, occurs alongside hyperleptinemia, suggesting to be related to leptin resistance as well (Lin et al., 2000; Scarpace and Zhang, 2009; Lindholm et al., 2013). Leptin receptors are ubiquitously expressed in the brain, and alterations in circulating leptin levels may affect its function in specific brain regions. Despite higher values of circulating leptin, its levels in cerebrospinal fluid (CSF) appear to be lower in obesity, suggesting impaired transport across the BBB and a mechanism for leptin resistance (Farr et al., 2015). This phenomenon could potentially be attributed to a restricted transfer of circulating leptin across the BBB. This proposition is underscored by research in obese mice, which indicates that the movement of leptin through the BBB becomes saturated, possibly serving as an avenue for the emergence of leptin resistance (Banks et al., 1999). The same team found out later that this was promoted by one prevailing hallmark of obesity, hypertriglyceridemia (Banks et al., 2004).

Crucially, leptin is required for the proper performance of the immune system since its absence has been associated with more infection-related deaths (Iikuni et al., 2008), less circulating CD4^+^ T cells and impaired T cell proliferation (Farooqi et al., 2002). The presence of leptin receptors on immune cells such as CD4^+^, CD8^+^ (Martín-Romero et al., 2000; Papathanassoglou et al., 2006), regulatory T cells (Treg) (De Rosa et al., 2007), natural killer cells (Zhao et al., 2003), as well as in monocytes/macrophages (Papathanassoglou et al., 2006) denote the influence that this cytokine has on the immune system.

On the other hand, adiponectin is inversely related to adiposity. Under normal BMI values, this adipokine has been shown to exhibit insulin-sensitizing, anti-inflammatory, anti-apoptotic, anti-atherosclerotic, as well as neuroprotective properties, such as the induction of neuronal progenitors (Pérez-González et al., 2011; Letra et al., 2019), which is reversed in obesity. Adiponectin reduction can be explained by a suppressive influence on adiponectin expression, through mechanisms such as DNA methylation. In adipocytes, DNA hypermethylation at specific regions of the adiponectin promoter, such as the R2 region, is orchestrated by enzymes like DNA methyltransferase 1. This epigenetic modification induces the formation of heterochromatin structures that downregulate adiponectin gene expression in the context of obesity (Kim et al., 2015).

Several studies have also demonstrated that anti-aging gene Sirtuin 1 (SIRT1) expression and consequently its function is regulated as part of inflammatory response. Interestingly, SIRT1 is altered by obesity and unhealthy diets. This protein is a NAD^+^-dependent deacetylase that mediates metabolic responses to nutrient availability (Sauve et al., 2006). In healthy conditions, contributes positively to liver lipid metabolism by restraining hepatic lipogenesis, promoting fatty acid β-oxidation, and ensuring the stability of cholesterol and bile acid levels (Kemper et al., 2013). Several other beneficial functions include cell survival, DNA repair, chromatin remodeling, and neuronal survival and differentiation (Yamamoto et al., 2007; Duan, 2013; Giblin et al., 2014). Upon obesity, SIRT1 is known to be repressed both in mice (Chalkiadaki and Guarente, 2012) and humans (Pedersen et al., 2008; Costa Cdos et al., 2010). It was demonstrated that in underdeveloped countries, the urbanization and the adoption of western diets lead to the dysregulation of SIRT1 due to changes in transcriptional regulators and modifications in chromatin. This dysregulation contributes to endocrine abnormalities, such as insulin resistance, non-alcoholic fatty liver disease, and disruptions in energy balance (Martins, 2013), which therefore triggers mitochondrial apoptosis, as well as alterations in the immune system (Yoshizaki et al., 2010; Martins, 2016, 2017).

Recent research has also highlighted the multifaceted roles of neurotrophins like nerve growth factor (NGF) and brain-derived neurotrophic factor (BDNF), traditionally known for their involvement in nerve growth and survival as well as their influence in various non-neuronal cell types and metabolic processes. These neurotrophins have been designated as metabotrophins (metabokines) due to their effects on glucose, lipid, and energy homeostasis. Studies have shown decreased levels of NGF and BDNF in conditions like metabolic syndrome, acute coronary syndromes, and obesity, suggesting their potential involvement in the development of atherosclerosis and metabolic disorders (Tore et al., 2008; Yanev et al., 2013; Frohlich et al., 2021). Moreover, adipose tissue has been identified as one of the different sources of NGF and BDNF, which may play a role in regulating metabolism and immune responses. Alongside NGF and BDNF alterations, hyperleptinemia and an increased number of mast cells in subcutaneous abdominal adipose tissue are also reported (Sornelli et al., 2007; Tore et al., 2008), shedding light on the role of these metabotrophins in the pathogenesis of obesity and related diseases. Genetic mutations impacting BDNF or its receptor TrkB result in significant overeating and severe obesity in both humans and mice (Yeo et al., 2004; Gray et al., 2006, 2007). BDNF gene expression is influenced by various factors like nutritional status, glucose levels, and anorexigenic hormones such as leptin and melanocortin, particularly in brain regions associated with appetite control like the ventromedial hypothalamus and dorsal vagal complex (Xu et al., 2003; Unger et al., 2007). This suggests that BDNF actively contributes to the regulation of satiety (Xu and Xie, 2016).

### 2.2. Obesity-derived CNS complications

#### 2.2.1. CNS inflammation

The chronic overconsumption of foods that are high in carbohydrates and saturated lipids in people with obesity can have a significant impact on cerebral glucose metabolism and functions, affecting insulin secretion, thereafter, being identified as one of the factors underlying the pathogenesis of neurodegenerative diseases (Biessels et al., 2006; de la Monte, 2012). Obesity induces not only metabolic complications in energy metabolism-related organs but also low-grade chronic inflammation throughout the body, including the CNS. Adipose dysfunction has been linked to altered brain metabolism, BBB disruption, neuroinflammation, neuronal dysfunction, brain atrophy, impaired mood, and cognitive decline (Luppino et al., 2010; Gustafson, 2012; Arshad et al., 2018). Due to the chronic inflammation produced by adipocytes, the exacerbated release of proinflammatory adipokines/cytokines to the bloodstream can be a facilitator of leukocyte infiltration into the CNS through the BBB, favoring the development of neurodegenerative diseases (Buckman et al., 2014; Davanzo et al., 2023).

Despite CNS having a distinct microenvironment, protected, and maintained in strict conditions mainly through the BBB, pathological features of obesity, such as hyperglycemia and a diabetic state, can disturb insulin transporters at the BBB (Price et al., 2012). Once the BBB is compromised, the CNS becomes vulnerable to external factors, such as the invasion of peripheral inflammatory cytokines increased during obesity, resulting in reduced synaptic plasticity and impaired neurogenesis (Kiliaan et al., 2014; Zheng et al., 2016). Signs of higher CNS inflammation can be seen through increased expression of CD45, a microglia marker, and glial fibrillary acidic protein, an astrocytic marker, along with higher values of cytokines such as TNF-α, IL-1β, and IL-6 levels in HFD mice (Thirumangalakudi et al., 2008). Indeed, both CNS resident immune cells showed activation in the presence of saturated FA or free FA (Patil et al., 2007; Wang et al., 2012; Martin-Jiménez et al., 2017). Moreover, high levels of TNF-α can block intracellular signaling by affecting insulin receptor substrate 1, having an essential role in neural health through many downstream pathways, such as phosphatidylinositol-4,5-bisphosphate 3-kinase (PI3)/Akt cascade (De Felice and Ferreira, 2014).

Different brain regions are subjected to obesity-induced neuroinflammation, such as the cerebral cortex, brainstem, hypothalamus, hippocampus, and amygdala. Thaler et al. (2012) found that inflammatory signaling in the hypothalamus was evident in rats and mice within 1–3 days of being subjected to a HFD, even before a substantial weight gain. Reactive gliosis and neuronal injury markers were also observed in the hypothalamic arcuate nucleus of rats and mice within the first week of HFD feeding. As in rodents, increased gliosis was found in the mediobasal hypothalamus of obese humans, assessed by magnetic resonance imaging (MRI), suggesting that obesity is associated with neuronal injury in a brain area crucial for body weight control (Thaler et al., 2012). Additionally, mice fed with HFD showed increased age-related oxidative damage, deeply associated with inflammation, particularly with a decline in NF-E2-related factor 2 levels and activity, which is responsible for protecting the brain against oxidative damage (Morrison et al., 2010).

#### 2.2.2. CNS mitochondrial dysfunction

Another prominent feature is mitochondrial dysfunction. Although the brain only represents 2% of the body’s total weight, it does present high energy demand, around 20% of total ATP produced within the body (Jain et al., 2010). Therefore, the mitochondrial role in the brain is fundamental. Once more, it has been demonstrated that obesity and excess energy intake shift the balance of mitochondrial dynamics and metabolic deterioration, leading to insulin resistance (Jheng et al., 2012). Accumulating evidence has shown that HFD can disturb mitochondrial function, reducing its oxidative capacity in the brain cortex and synaptosomal fraction. The alterations caused by HFD had an adverse effect on the BDNF pathway in the brain cortex, crucial for synaptic plasticity and energy metabolism. Furthermore, HFD led to a reduction in mild uncoupling in brain mitochondria, which functions to keep mitochondrial membrane potential under the threshold required for reactive oxygen species (ROS) generation (Cavaliere et al., 2019).

Increments in lipid peroxidation, ROS production, and cytochrome c oxidase usage, contrasting with a reduction in FA oxidation and ATP production are some features underlying mitochondrial dysfunction. All these factors contribute to an overall decrease in brain performance, exhibiting a correlation with cognitive decline (da Silva and Ferrari, 2011). This impairment is even more pronounced when looking at the synaptic regions where neuronal plasticity becomes endangered, contributing to neurotransmission blockade and cognitive failure associated with neurodegenerative diseases (Reddy and Beal, 2008; Merlo et al., 2016; Devine and Kittler, 2018).

#### 2.2.3. Gut-brain axis deregulation

An emerging field of study has been showing the impact that gut microbiota can have on the CNS and vice versa, commonly known as the gut-brain axis. The core pathways involved in this two-way communication comprise the central, enteric, and autonomic nervous systems, along with the hypothalamic-pituitary-adrenal axis (Carabotti et al., 2015). The appropriate diversity of microbiota is necessary for normal brain development and improved cognitive ability, being supported by nutrition and diet. Gut microbiota can regulate fat storage, as well as harvest energy from the diet (Turnbaugh et al., 2006), which can have an impact when obesity is present.

In mice fed with an HFD, gut microbiota diversity is strongly halted, in line with reduced synaptic plasticity, disruptions in exploratory and cognitive functions, and higher susceptibility to anxiety-like behavior (Daniel et al., 2014; Liu et al., 2015). Similarly, when fed with a diet rich in sucrose, the spatial bias for short-term and long-term memory, as well reversal training are compromised (Liu et al., 2015; Rogers et al., 2016). Surprisingly, Turnbaugh et al.’s (2006) team proved that obesity-associated metabolic phenotypes could be transmitted via intestinal microbiota alone in germ-free mice. A study in humans revealed similar results whereas fecal microbiota from twins discordant for obesity were transplanted to germ-free mice fed on a low-fat diet. Mice that received gut microbiota from the obese twin found their total body and fat mass increased, as well as the development of obesity-associated metabolic phenotypes. Remarkably, this phenotype can be reversed following co-housing with mice transplanted with the lean microbiota (Ridaura et al., 2013). Furthermore, obesity-associated gut dysbiosis has been linked to the release of various bacterial toxins into the bloodstream, which can exert an influence on the CNS (Breton et al., 2022). An illustrative example is lipopolysaccharide (LPS), which, under these conditions, has been reported to affect microglial cells. This occurs by LPS binding to the TLR4/CD14 complex on peripheral monocytes/macrophages or brain microglia, subsequently activating NF-κB and promoting the production of cytokines such as IL-1, IL-6, and TNF-α (Sharma and Martins, 2023).

#### 2.2.4. Cognitive impairment and brain atrophy

As a result of CNS homeostasis disruption, cognitive function can easily be compromised. One factor is the disturbance of cerebral blood flow (CBF) since it is extremely important for the proper delivery of essential nutrients and oxygen to the brain. Cerebral hypoperfusion, the most common CBF disruption, can occur due to endothelial dysfunction induced by obesity, which decreases nitric oxide synthesis and therefore increases oxidative stress production (Toda et al., 2014). Triggering receptor expressed on myeloid cells 2 (TREM2) and PI3/Akt cascade pathways were described to be involved in these processes (Shu et al., 2013; Zhang et al., 2020).

Overall, all anthropometric measures of obesity, such as body weight, BMI, or waist circumference (WC) show a negative association with cognitive performance (Elias et al., 2012), and worse executive function (Reinert et al., 2013). Gray matter CBF specifically is directly correlated with cognitive function, and in obesity, higher values of BMI were linked with lower levels of gray matter CBF. Associated with cerebral hypoperfusion are vascular cognitive impairment and dementia (Yu et al., 2022), indicating that cognitive function can be compromised without a regular CBF. Moreover, memory performance is strongly reduced with obesity, such as delayed recall and recognition (Cournot et al., 2006; Gunstad et al., 2006) along with visual what-where-when episodic memory tasks (Cheke et al., 2016). DIO models in rodents demonstrate impairment in working memory and learning (Kanoski and Davidson, 2010; Jurdak et al., 2013), which is attributed primarily to inflammation and impaired insulin action in the brain (De Felice and Ferreira, 2014). Insulin plays a crucial role in the modulation of synaptic plasticity, behavior, learning memory, and cognitive functions (Zhao et al., 1999; Cholerton et al., 2016). Moreover, insulin receptors and insulin-sensitive glucose transporters are widely distributed in the brain (Havrankova et al., 1978; Sara et al., 1982; Wickelgren, 1998) and highly concentrated in regions such as the cortex and the hippocampus that support memory formation and learning, suggesting that insulin is important for maintaining normal cognitive function (Watson and Craft, 2003, 2004; Shapiro et al., 2014). There is further evidence suggesting a connection between obesity and cognitive function through alterations in DNA methylation of memory-associated genes, particularly SIRT1 within the hippocampus. In mice with a specific knockout of SIRT1 in forebrain neurons exhibited memory deficits similar to those observed in obese mice. This aligns with the hypothesis that the reduction of hippocampal SIRT1 due to a HFD may contribute to memory impairment associated with obesity (Heyward et al., 2016).

Brain structure integrity (both white and gray matter) is also believed to be compromised in obesity due to impaired CBF, leading to ischemic stress and therefore neuronal damage (Bobb et al., 2014). Several studies have demonstrated that brain volume declines as a function of obesity (Gustafson et al., 2004; Ward et al., 2005; Pannacciulli et al., 2006). Raji et al. (2010) have also shown a strong relation between obesity and brain atrophy in a cognitively normal elderly population. In line with these studies, experiments using proton magnetic resonance spectroscopy showed that higher BMI lowers neuronal viability in several brain regions including frontal, parietal, and temporal lobes (Gazdzinski et al., 2010). Moreover, most of the studies demonstrate a negative correlation between BMI and gray matter volume/integrity (Weise et al., 2013; Veit et al., 2014; Janowitz et al., 2015; Shaw et al., 2017; Hamer and Batty, 2019; Nota et al., 2020). Curiously, there is a divergence of results regarding the relationship between obesity and white matter volume/integrity. One study presented a positive correlation between the levels of free FA in the bloodstream and the volume of white matter in the left temporal and occipital lobes of individuals with obesity. This observation suggests that the observed variations in white matter within the context of obesity could potentially be attributed to irregular lipid metabolism and the accumulation of lipids within the brain. However, it is important to note that the increase in white matter volume in obesity is not necessarily linked to fat accumulation in the brain (Haltia et al., 2007).

**FIGURE 1:**
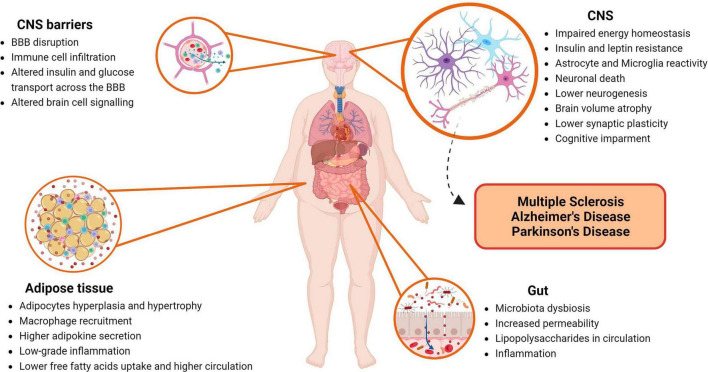
Impact of obesity on the central nervous system (CNS). This figure highlights major players and disturbances within the human body that contribute to neurodegeneration of the CNS induced by obesity. BBB, blood-brain barrier; CNS, central nervous system. Created with BioRender.com.

In this chapter, we explored the multifaceted ways through which obesity can impact the CNS, leading to neurodegeneration. A concise visual summary of these influential factors and disturbances, can be depicted in Figure 1. This figure succinctly summarizes the core themes of our discussions, offering a comprehensive overview of the intricate relationship between obesity and CNS health.

## 3. Multiple sclerosis

Multiple sclerosis is the most prevalent neurological condition affecting young adults, particularly women, reaching 2.9 million people worldwide and often develops between the ages of 20 and 40 (The Multiple Sclerosis International Federation, 2020)^2^. The clinical symptoms include sensorimotor defects, visual disturbances, ataxia, fatigue, difficulties in thinking, and emotional problems (Compston and Coles, 2008). As a chronic autoimmune disease, is characterized by inflammation, demyelination, and neurodegeneration of the CNS. Myelinated axons within the CNS are targeted, leading to varying degrees of myelin and axon destruction, and resulting in significant physical disability. These localized lesions are believed to arise from the infiltration of immune cells, which encompass T cells, B cells, and myeloid cells, into the CNS tissue, giving rise to associated tissue injury (Filippi et al., 2018). MRI is highly effective in identifying demyelinating lesions, serving a dual purpose: assisting in diagnosis, along with other procedures, as well as for disease monitoring accompanied by the expanded disability status scale (EDSS), a method to quantify disability in MS (Barkhof, 1999). Regarding disease activity, this can be defined in three main clinical forms. Most cases start with reversible neurological episodes (relapses), lasting days to weeks, seen in clinically isolated syndrome (CIS) or in relapse-remitting MS (RRMS). Frequently, in time, permanent neurological issues and disability emerge, being converted into secondary progressive MS (SPMS). A minority possesses a continuously progressive form, since onset, termed primary progressive MS (PPMS) (Lublin et al., 2013).

Since MS has a multifactorial background of potential causes, the etiology is not yet identified, but genetic susceptibility and environmental risk factors play a significant role in raising the likelihood of developing MS. Obesity is one of the players involved in both risk for MS development and respective progression, although it is unclear how obesity may alter the course of the disease (Filippatou et al., 2020). Nutrition’s direct role in MS development also lacks concrete evidence. However, an early study performed in the 1950’s showed a positive correlation between the higher consumption of animal fat coupled with lower fish intake and the onset of MS (Swank et al., 1952). More recent epidemiological studies indicate that diets high in saturated fat intake, as well as in carbohydrates and low in polyunsaturated fats may be linked to higher MS prevalence (Riccio, 2011; Esposito et al., 2018). Moreover, currently, there is no cure for MS, but certain lifestyle changes and disease-modifying treatments (DMTs) can help reduce the extension of the damage and the occurrence of the relapses, preventing or retarding the worsening of the disability over time.

### 3.1. Obesity is a risk factor for MS development and progression

Significant changes have already been observed at the onset and progression of MS, in the presence of obesity. Higher BMI and/or WC have been linked not only with higher odds of developing MS but in fact with a higher risk of conversion from CIS to MS, higher relapse rates, more severe disability in time, higher cognitive decline, and brain atrophy (Stampanoni Bassi et al., 2019; Filippatou et al., 2020; Fitzgerald et al., 2020; Lutfullin et al., 2022). Interestingly, one study used BMI as a disease progression predictor and found that obesity was associated with a faster conversion from CIS to MS along with a higher relapse rate (Manuel Escobar et al., 2022). One study evaluated through the course of 5 years, different parameters, such as the EDSS, MRI new lesions, and relapse rates in MS patients comparing obese with non-obese individuals. MS obese patients had an 8-fold higher chance of experiencing at least a 1-unit increase in EDSS than patients with normal weight. Concerning CNS lesions and relapse rate (chance of having at least 1 relapse within 5 years), the odds were 2.6 and 3.8 times higher, respectively, in obese subjects than in non-obese ones (Ben-Zacharia, 2015).

As a risk factor for MS development, obesity has been mentioned to play a role early in life, during childhood, and especially in adolescence, even if MS onset manifests several years later (Hedström et al., 2014, 2016). Likewise adult-onset, obesity is also associated with the pediatric onset of MS (POMS) (Langer-Gould et al., 2013). In fact, the relationship between BMI and initial inflammation in the CSF of prepubertal patients with POMS suggests that there may be a connection between high BMI, sexual hormones, and the onset of POMS (Milles et al., 2021). Langer-Gould et al.’s (2013) team found out that, especially in girls, there was a significantly increased risk of developing MS or CIS, being odds 1.58, 1.78, and 3.76 for overweight, moderate obesity, and extremely obese, respectively. Elevated susceptibility to POMS seemed to correlate with obesity, and individuals classified as obese exhibited suboptimal reactions to initial therapeutic interventions. The potential factors influencing treatment responsiveness appeared to center around modified pharmacokinetics (Huppke et al., 2019).

In addition, there is evidence that obesity potentiates MS susceptibility by interacting with other MS risk-associated genetic and environmental elements such as human leukocyte antigen variants (Hedström et al., 2014), as DRB1*1501 (Hayes and Ntambi, 2020), and Epstein-Barr virus infection (Hedström et al., 2015). Through this process of potentiation, there is a variation in the combined risk values that exceeds the simple sum of individual risk factors, leading to an increased overall risk estimation (Gianfrancesco and Barcellos, 2016).

Regarding the various DMTs indicated for MS, individuals with higher BMI have presented inferior responses to these medications than non-obese individuals. Kvistad et al. (2015) showed a negative correlation between BMI and IFN-β treatment efficacy. They concluded that BMI should be considered when assessing the results of this therapy in MS patients, as well as taking steps for weight reduction during treatment as a way of improving the outcome (Kvistad et al., 2015). A team from the King Abdulaziz University Hospital (Saudi Arabia) found similar results when exploring this effect in various DMTs, including IFN-β, fingolimod, ocrelizumab, and rituximab. A positive relation was found between BMI and the number of relapses occurring at least 6 months following the start of each DMT. Overweight patients had a worse course than normal and underweight patients, and an increase in BMI appeared to be related to a lower response to DMTs. Once more, the most likely causes of variation in DMT responsiveness are pharmacokinetic differences (Aljehani et al., 2023).

Although the specific mechanism by which obesity contributes to MS onset and progression is not clear yet, researchers have demonstrated several detrimental effects of obesity on the pathogenesis of this disease, focusing on the interplay between obesity and immune cells, inflammation, adipokines, and other immune-mediated cytokines.

### 3.2. Obesity-related mechanisms that contribute to MS onset and progression

#### 3.2.1. Brain volume decline

Brain volume decline has been reported both in MS patients, as well as in obese individuals even without MS as described before (Gustafson et al., 2004). When these factors are combined, this effect can be further amplified. In obese MS patients was found an interesting negative correlation between the number of blood pro-inflammatory monocytes and the brain volume, which indeed resulted in a worse disease course. This correlation is thought to be due to ceramide-induced DNA methylation of anti-proliferative genes, resulting in decreased expression levels that favor proliferation (Castro et al., 2019). Additionally, other studies have suggested that obesity in MS patients may also be associated with increased rates of retinal neuroaxonal loss, as a marker of neurodegeneration in MS (Costello and Petzold, 2020).

Lower CBF, as well as less oxygen and nutrients to the brain, seen in obesity (Selim et al., 2008; Willeumier et al., 2011), contribute to an increased oxidative stress state. Interestingly, oligodendrocytes, one of the most affected cells in MS are also one of the most sensitive type of cells to oxidative stress. Oxidative stress, produced by HFD, promotes lower mitochondrial function and differentiation in these cells, as well as the exhibition of apoptotic markers which might accelerate neuro-axonal loss and more profound neurodegeneration in time. Furthermore, their progenitors (oligodendrocyte progenitor cells) are also subjected to apoptosis in these conditions, compromising even further the remyelination process due to their failure to reach maturity and myelinate in these conditions (Langley et al., 2020).

#### 3.2.2. Neuroinflammation and BBB disruption

Neuroinflammation can be obesity-derived, including BBB breakdown as one significant feature (Davanzo et al., 2023). In the most used mouse model of MS, the experimental autoimmune encephalomyelitis (EAE) model, mice under HFD presented signs of CNS inflammation and disease severity exacerbation. Analysis of immune infiltrates at the peak of the disease showed that the HFD-fed group had higher levels of pro-inflammatory monocytes, macrophages, and IFN-γ^+^ CD4^+^ T cells, as well as white matter lesions in the spinal cord and BBB disruption. These findings suggest that obesity-induced neuroinflammation can promote BBB disruption, allowing the infiltration of immune cells and the activation of microglia, further exacerbating CNS inflammation and consequently disease progression (Davanzo et al., 2023). Likewise, a different study indicated an aggravation of EAE in mice fed with HFD being linked to an intensified microglial activation and greater proliferation of T helper cell (Th)1 and Th17 cells (Ji et al., 2019). Using short-chain fatty acid propionate, a group of researchers were able to increase Treg functionality while lowering Th17 cell activity in EAE-induced mice under HFD. This resulted in a reduction of the severity of EAE, accompanied by decreased demyelination and fewer immune cell infiltrations to the spinal cord. In the same study, a similar restoration of this Th17/Treg ratio was also observed in MS patients upon propionic acid intake (Haase et al., 2021).

It is noteworthy that these alterations in the inflammatory state, observed in mice, appear to be time-dependent, both for the age of animals tested, as well as the duration of diet consumption (Guillemot-Legris and Muccioli, 2017). Interestingly, likewise in mice models, the relationship between obesity and MS risk in humans is time-dependent, specifically during childhood and adolescence (Hedström et al., 2014).

#### 3.2.3. Leptin and adiponectin

Leptin has garnered considerable interest due to its levels being significantly elevated in obesity and linked with a more proinflammatory state, which has negative effects on MS (Matarese et al., 2010). The receptor for this cytokine (LepR) has been found to be overexpressed both in CD8^+^ T cells, as well as in monocytes during the acute phase of the disease (Frisullo et al., 2007) and leptin has been found at high levels in CNS lesions of MS *post-mortem* samples (Lock et al., 2002). In MS patients, it has been demonstrated that leptin levels are directly correlated with CSF IFN-γ secretion and negatively correlated with the proportion of circulating Treg cells (Matarese et al., 2005). A study analyzed individuals under 20 years old and found that higher leptin levels were independently associated with an increased risk of MS (Biström et al., 2021). Another study evaluated the effect of leptin on T cells in MS patients, having demonstrated an increase in autoreactive T cell proliferation and proinflammatory cytokine production, in opposition to the suppression of Treg cells (Marrodan et al., 2021).

Interestingly, it was demonstrated that leptin deficiency due to acute starvation, leads to delayed onset of EAE, and attenuated clinical scores (Sanna et al., 2003). In a similar approach through leptin neutralization, EAE clinical symptoms were also reduced (De Rosa et al., 2006). Moreover, leptin-deficient animals exhibit lower CNS inflammation and resistance to EAE induction, while leptin replacement was able to reestablish EAE susceptibility (Matarese et al., 2001).

On the other hand, adiponectin, an adipokine with anti-inflammatory effects, has been shown to play a protective role in EAE, since adiponectin-deficient mice have demonstrated to develop worse EAE with greater CNS inflammation, demyelination, and axonal injury compared to wild-type mice (Piccio et al., 2013).

#### 3.2.4. Gut microbiota disturbances

The relationship between obesity, gut microbiota, and MS is increasingly being seen as a subject to be further explored. New findings discuss the impact of obesity on gut microbiome dysbiosis, as well as the associated metabolic pathways and intestinal permeability, in fostering EAE disease severity (Shahi et al., 2022). The gut microbiota of EAE mice displays changes, including an elevated presence of *Proteobacteria* and *Desulfovibrionaceae* bacteria, increasing sulfur metabolism, as well as lipopolysaccharides and long-chain FA biosynthesis which are linked with inflammation. The elimination of gut microbiota in EAE mice resulted in a reduction in disease severity, highlighting the critical role of gut bacteria in MS (Shahi et al., 2022). Furthermore, using a fructose-rich diet in EAE mice led to a significant impact on gut bacteria, decreasing beneficial bacteria while increasing the ones that could potentially cause inflammation. Immune modulation was also verified, although, only subtle changes were observed in EAE severity (Peterson et al., 2023).

Overall, obesity is linked to higher risk and progression of MS, influencing relapse rates, brain volume decline, neuroinflammation, hormone levels (leptin and adiponectin), and gut microbiota. This connection seems to start early and affect treatment responses.

## 4. Alzheimer’s disease

Alzheimer’s disease is the most prevalent type of dementia in older individuals, with a worldwide estimate of 24 million cases. Without more effective treatments to tackle this disease, the number is expected to quadruple by 2050 (Reitz and Mayeux, 2014). The first manifestations of the disease comprise memory loss which then progresses to various cognitive impairments and ultimately leads to death. One of the concerns with this disease is that the imperceptible ongoing pathology can begin to form up to two decades before the first symptoms appear (Bateman et al., 2012). AD is characterized by neuronal and synapse loss, especially in the cerebral cortex, and with a greater impact on the hippocampus (Mu and Gage, 2011; Nisticò et al., 2012; Hollands et al., 2016). β-amyloid peptide (Aβ_1–42_) and neurofibrillary tangles accumulation from the hyperphosphorylation of tau protein are hallmarks of AD pathology which are directly linked with neuroinflammation (Caruso et al., 2019).

### 4.1. Obesity is a risk factor for AD development and progression

Among other risk factors, obesity is known to have a link with a higher risk for cognitive decline, dementia, and AD onset (Povova et al., 2012; Baumgart et al., 2015; Anjum et al., 2018; Rasmussen Eid et al., 2019). Research has suggested that higher BMI in mid-life may contribute to the underlying mechanisms for AD and cognitive decline (Whitmer et al., 2005, 2008; Beydoun et al., 2008). However, in later life, the relationship between BMI and AD appears to go against this trend, with some studies indicating that higher BMI may diminish the burden or aggressiveness of the disease (Hughes et al., 2009; Kim et al., 2016).

Some theories have proposed a potential connection between dietary elements and the onset of AD. These factors include both a deficiency and/or an excess of dietary compounds. High fat diets and excessiveness of saturated FA were already linked with hyperinsulinemia, which in turn is associated with higher risk of AD (Luchsinger et al., 2004; Luchsinger and Mayeux, 2004; Laitinen et al., 2006). On the other hand, deficiency in vitamins B6 and B12, E and C were also associated with higher risk for AD (Mecocci and Polidori, 2012; An et al., 2019; Mielech et al., 2020) since these relate to the diminishing of lipid peroxidation and oxidative stress induced by β-amyloid and inhibition of inflammation signaling pathways (Butterfield et al., 2002; Luchsinger and Mayeux, 2004). Interestingly, a western HFD alone can impair cognitive function in WT mice (Hao et al., 2016; Cifre et al., 2018), and therefore even worse outcomes in AD mouse models (Sah et al., 2017; McLean et al., 2018). Obesity shares a range of metabolic changes with AD. These conditions are linked to cognitive decline, accompanied by alterations in lipid metabolism, CNS and peripheral inflammation, and altered levels adipokines (for NGF and BDNF, see Yanev et al., 2013; Frohlich et al., 2021), as well as the interaction with genetic factors such as the ApoE4 allele (Reitz and Mayeux, 2014; Jones and Rebeck, 2018).

### 4.2. Obesity-related mechanisms that contribute to AD

#### 4.2.1. Amyloid-β disturbances

Prolonged consumption of HFD in rodents alone has been shown to result in increased Aβ precursor protein (APP) levels both in the hippocampus (a crucial region for learning and memory formation), as well as in adipose tissue, along with an active state of inflammation (Puig et al., 2012). A similar study found an increase in CNS Aβ levels, which were linked to the increase of APP and β-site APP-cleaving enzyme 1. These changes are often accompanied by oxidative stress and mitochondrial dysfunction, further exacerbating the pathological effects of Aβ accumulation (Nuzzo et al., 2015).

As a result of the altered metabolic patterns caused by obesity, the creation and buildup of advanced glycosylation end-products (AGEs) and their precursors can be triggered. AGEs are harmful substances that can cause damage to the CNS by promoting the clustering of Aβ through a process called glycation (Vitek et al., 1994). This is due to the capacity of receptors that bind to AGEs that can also bind to Aβ (Srikanth et al., 2011). Unsurprisingly, in several strains of transgenic mice models of AD, DIO has been observed to increase the levels of Aβ (Ho et al., 2004; Julien et al., 2010; Kohjima et al., 2010; Barron et al., 2013; Moser and Pike, 2017), as well as tau phosphorylation (Julien et al., 2010; Leboucher et al., 2013; Mehla et al., 2014; Takalo et al., 2014).

While empirical evidence on the possible relationship between obesity, cholesterol, and Aβ production is currently lacking, it is worth noting that this element is known to be able to cross the BBB and to have a high affinity for APP and Aβ (Harris and Milton, 2010). Interestingly, β- and γ-secretases are primarily situated within lipid rafts abundant in cholesterol, indicating that higher cholesterol levels could potentially impact their functioning and thereafter induce the amyloidogenic pathway (Grimm et al., 2007; Picone et al., 2020).

#### 4.2.2. Oxidative stress and energy unbalance

In the context of AD, it is worth mentioning that oxidative stress is one of the hallmarks of the disease and is considered one of the disease-triggering mechanisms (Finder, 2010), and its levels are further increased in the presence of obesity. Obesity-related energy balance changes are associated with the loss of synaptic contacts, as well as memory problems in 3xTgAD mice, a mouse model of AD (Knight et al., 2014). Corroborating that, another group demonstrated that the same strain under HFD has memory problems associated with a reduction in antioxidant enzymes (heme oxygenase-1 and manganese-dependent superoxide dismutase) due to inhibition of Akt/nuclear factor erythroid 2-related factor 2 signaling pathway (Sah et al., 2017). 5XFAD mice, another mouse model of AD, also under HFD, showed signs of cognitive impairment and hippocampal oxidative stress (Lin et al., 2016). Furthermore, in APP/PS1 AD mouse model under HFD, the results showed an increased inflammatory response and Aβ monomers and plaques, as well as exacerbated behavioral deficits, especially in sensory-motor function (Bracko et al., 2020).

#### 4.2.3. Brain atrophy

Some studies have shown that brain volume loss linked to being overweight or obese can occur in the same areas as those affected by AD pathology, particularly in the hippocampus. These alterations have been observed in individuals with normal cognitive function, as well as those with mild cognitive impairment or AD (Ho et al., 2010; Raji et al., 2010; Boyle et al., 2015). Throughout adulthood, higher BMI is associated with reduced perfusion in brain single photon emission computed tomography scans during both rest and concentration states. This included the hippocampus region, the most affected in AD, indicating a negative impact of obesity on brain function (Amen et al., 2020). Higher BMI and WC are associated with a reduction in cortical thickness, both within and outside regions targeted by AD pathology. This suggests that the impact of obesity on the brain can play a global role rather than specific to AD (Caunca et al., 2019). Additionally, there is evidence to suggest that increasing WC is linked to a decrease in gray matter volume (Janowitz et al., 2015).

#### 4.2.4. Leptin and adiponectin

There is growing evidence that leptin resistance plays a role in the development and progression of AD. In *post-mortem* samples from AD patients, a considerable number of cells that express LepR were found to be in the same location as the neurofibrillary tangles. This co-localization was linked to a reduction in the number of active, phosphorylated LepRs, possible due to the obstruction caused by neurofibrillary tangles to the interaction between circulating leptin in the brain and respective receptors, potentially causing leptin resistance in those neurons. This phenomenon could explain the observed increase in CSF leptin levels (Bonda et al., 2014). A similar study found concordant results both in *post-mortem* samples from AD patients, as well as in Tg2576 and apoE4 mouse models of AD. A decreased expression of LepR was found specifically in the hippocampus in the three conditions adding evidence to leptin resistance (Maioli et al., 2015).

Contrary to what happens in MS, in AD leptin seems to have a protective effect against the development of the disease by reducing the accumulation of Aβ through the activation of insulin-degrading enzyme (Marwarha et al., 2010). In addition, leptin was also reported to have a protective role in this disease by potentially modifying the lipid composition of membrane lipid rafts, leading consequently to a decrease in the activity of β-secretase in neuronal cells (Fewlass et al., 2004). Moreover, according to Doherty et al. (2013) and Pérez-González et al. (2014) studies, leptin prevents the negative effects of Aβ on hippocampal long-term potentiation (LTP) and long-term depression, restoring normal synaptic function and increasing synaptic density, as well as rescuing memory deficits. In another study using a rat model of AD, where Aβ was injected intracerebroventricularly, the chronic administration of leptin was able to restore spatial memory and late-phase LTP function (Tong et al., 2015).

Adiponectin has several beneficial effects on AD. One of the mechanisms is through the regulation of microglia activation, which is noteworthy since chronic inflammation caused by microglial cell activation has been described to induce AD and metabolic distress-related pathologies (Chabry et al., 2015; Nicolas et al., 2017). However, adiponectin levels are found altered both in plasma and in the CSF of AD patients. There is a tendency among studies concerning the increase of adiponectin levels in serum but the opposite in the CSF, possibly to counteract potential abnormalities in central signaling (Khemka et al., 2014; Ma et al., 2016; Waragai et al., 2016; Wennberg et al., 2016).

#### 4.2.5. Insulin resistance

Individuals with AD have been found to exhibit reduced expression of the insulin receptor, insulin-like growth factor 1 receptor, and insulin receptor substrate 1 in the hippocampus and hypothalamus. Furthermore, Willette et al. (2015) found increased amyloid deposition in the frontal and temporal areas of middle-aged humans with higher insulin resistance. When Tg2576 mice, an animal model of AD, were fed with HFD, they exhibited signs of obesity and insulin resistance, as well as a surge in Aβ production in the brain (Kohjima et al., 2010). Several mechanisms help explain the link between brain insulin resistance and AD. These include hyperinsulinemia, which leads to competition between insulin-degrading enzyme and Aβ, reducing Aβ brain clearance; binding of Aβ oligomers to the insulin receptors, causing impairment of the insulin signaling pathways; and downregulation and internalization of insulin receptors due to Aβ oligomers binding (Zhao et al., 2008; Di Carlo et al., 2012; Ng and Chan, 2017).

In summary, obesity’s association with AD involves complex interactions such as leptin resistance, insulin dysfunction, altered glucose metabolism, oxidative stress, amyloid-beta disturbances, and brain atrophy. Leptin and adiponectin also play roles, with potential protective effects in this disease.

## 5. Parkinson’s disease

Parkinson’s disease is a neurodegenerative disorder that affects the CNS, being the second most common neurodegenerative disease after AD, with an incidence of 1–3% for people over 65 years old, and is characterized by symptoms such as tremors, bradykinesia, rigid muscles, and impaired posture and balance (Maiti et al., 2017). One hallmark of PD is the degeneration of dopaminergic neurons in the nigrostriatal pathway, more specifically in the substantia nigra pars compacta (SN) and the occurrence of Lewy bodies within neurons, which contain accumulations of alpha-synuclein, neurofilaments, and ubiquitin (Spillantini et al., 1997). Several factors, including mitochondrial dysfunction (Xu et al., 2014; Hu and Wang, 2016), oxidative stress (Gaki and Papavassiliou, 2014), neuroinflammation (Wang et al., 2015a), excitotoxicity and iron deposition (Licker et al., 2009), have been suggested to contribute to the degeneration of the dopaminergic system in the CNS, leading to PD (Barreto et al., 2015). The generation of free radicals through oxidation, nitrosylation, and peroxidation is also associated with neuronal damage (Barreto et al., 2015). Many genes related to PD, such as *alpha-synuclein*, *Parkin*, and *Pten induced kinase 1 (PINK1)*, encode proteins that are essential for mitochondrial homeostasis. PINK1/Parkin-mediated mitophagy is one of the processes that contribute to mitochondrial quality control, with PINK1 detecting mitochondrial depolarization, ROS, and protein misfolding and initiating mitophagy. However, when disrupted, may hinder mitochondria’s ability to eliminate oxidized proteins, which could potentially lead to the mitochondrial dysfunction seen in PD (McLelland et al., 2014; Shirihai et al., 2015).

### 5.1. Obesity as a potential risk factor for PD development and progression

As seen in MS and AD, obesity has a harsh impact on brain homeostasis therefore it has also been linked to PD. As described before, in obese individuals, adipose tissue produces adipokines that upregulate systemic inflammation and cause insulin resistance, potentially accelerating the progression of PD (Greenberg and Obin, 2006). It is important to point out that although the effect that obesity should have on neurodegenerative diseases seems logical, in the case of PD it is still not so clear. There are several conflicting results regarding the relationship between body weight and PD. Some studies suggest that there is no significant relationship between BMI and the future development of PD (Logroscino et al., 2007; Palacios et al., 2011; Roos et al., 2018), while others indicate that being overweight may be a potential risk factor compared to a normal BMI (Chen et al., 2014). Interestingly, looking at the other end of the spectrum, a higher risk of developing PD was observed in underweight individuals (Reife, 1995; Harrington et al., 2009; Wang et al., 2015b). The impact of being underweight was more pronounced in individuals with diabetes mellitus (Wang et al., 2015b).

Concerning diets, most of the studies focus on a single nutrient approach. Cholesterol, oxysterols, and saturated FA have been implicated in PD pathogenesis, potentially affecting α-synuclein aggregation, dopaminergic neuron destruction, oxidative stress, and cytokine production (Liu et al., 2010; Bousquet et al., 2011; Fan et al., 2013; Doria et al., 2016; Erro et al., 2018). Cohort studies suggest a protective role for polyunsaturated fatty acids (PUFA), with ω-6 PUFA potentially exerting a negative effect and ω-3 PUFA and α-linolenic acid offering protection, possibly through their role in inflammation (Kamel et al., 2014). The impact of antioxidant vitamins remains uncertain, but vitamin E intake has been associated with reduced PD risk in some studies (Etminan et al., 2005).

### 5.2. Obesity-related mechanisms that contribute to PD

To better understand the effects of diet/body weight on PD, Kao et al. (2020) found that HFD causes neuroinflammation, with increased astrogliosis in the SN and striatum along with fewer dopaminergic neurons in the SN and decreased dendritic spine density on the striatum. These results were also linked with the downregulation of peroxisome proliferator-activated receptors in the substantia nigra and ventral tegmental area, which can reduce neuroinflammation, oxidative stress, and dysfunction of mitochondria and peroxisomes within the CNS. In addition, these mice exhibited symptoms characteristic of PD, including cognitive impairment, increased anxiety, and reduced mobility (Kao et al., 2020).

A different study focused on tyrosine hydroxylase (TH), a precursor molecule for dopamine synthesis. By using an HFD-induced obesity model, their results showed a reduction in TH levels and an elevation in TH phosphorylation in the ventral tegmental area, which was not linked with α-synuclein, but with obesity-induced insulin resistance, inflammation, oxidative stress, and activation of astroglia and microglia. In terms of behavioral studies, the results were similar to Kao et al.’s (2020) study (Bittencourt et al., 2022).

Collectively, the impact of obesity on PD is not straightforward and may vary depending on individual factors and underlying mechanisms. Further research is needed to better understand the complex interplay between obesity and PD.

## 6. Diet and exercise as rescuing intervention

In recent times, there has been a growing recognition of the significant role that dietary and physical activity interventions play in the modulation of obesity and consequently in CNS disease activity (Martins, 2015).

Among various dietary patterns, the Mediterranean diet emerges as one of the greatest diets when it comes to reducing the risk of neurodegenerative disorders. This diet, characterized by long-term consumption of plant-based foods, grains, legumes, fish and, olive oil as a primary source of fat, and a moderate intake of red wine, stands out for its potential in chronic disease prevention. The molecular mechanisms underlying the protective effects of the Mediterranean diet are attributed to its abundant content of antioxidants, polyphenols, monounsaturated, and polyunsaturated fatty acids (Franco et al., 2023). Nevertheless, additional research is required to identify the key elements within the Mediterranean diet that contribute most significantly to this protective role, and determine the specific stages of life when this diet may exert its most pronounced effects.

The Mediterranean-DASH Intervention for Neurodegenerative Delay (MIND) diet, is a hybrid diet that combines elements from both Mediterranean diet and the Dietary Approaches to Stop Hypertension (DASH) diet, focusing on neuroprotective dietary components. The DASH diet is similar in many ways to the Mediterranean diet, although it places more emphasis on fat-free or low-fat dairy and meat products and includes more whole grains. A research study using this hybrid diet involved 960 participants over nearly 5 years and found that a higher MIND diet score was associated with a significantly slower decline in overall cognitive function and performance in specific cognitive domains (Morris et al., 2015).

Another popular diet is the ketogenic diet. This dietary strategy is characterized by its emphasis on high fat intake and low carbohydrate consumption. Its main purpose is to trigger a metabolic state known as ketosis, during which the body primarily utilizes fat for energy rather than carbohydrates. Note that, aside from its established use for epilepsy, and non-neurological conditions like heart disease, diabetes, obesity, autism, glioblastoma, and certain types of cancer, the ketogenic diet is not yet advised as a recommended approach for alleviating symptoms or slowing down the progression of any neurological diseases. Nevertheless, recent studies have shown promising results concerning neurodegenerative neurological diseases (Gough et al., 2021).

In MS, dietary intervention can lead to a reduction in neurological fatigue, disability scores (Katz Sand et al., 2019; Ovcharova et al., 2022), as well as depression (Yu et al., 2023). Different studies reported that Mediterranean diet adherence was associated with improved quality of life and lower disability levels as well as fatigue (Katz Sand et al., 2019, 2023; Uygun Özel et al., 2023). The MIND diet has also been reported to be associated with reduced odds of MS development suggesting it to be a beneficial approach for preventing MS (Noormohammadi et al., 2022). Moreover, the ketogenic diet is also considered to be one of the healthier diets, safe and tolerable over a 6-month period. Participants experienced significant improvements in various aspects, including reductions in fat mass, decreased self-reported fatigue and depression, increased quality of life (both physical and mental health), improved neurological disability, and positive changes in adipose-related inflammation markers (Brenton et al., 2022). Physical activity also presents an advantageous rehabilitation approach, aiding in symptom management, functional recovery, enhancement of quality of life, and the fostering of overall well-being (Motl et al., 2017). Despite the promising outcomes observed with dietary and physical interventions, the level of patient engagement remains suboptimal among individuals with MS (Motl et al., 2017; Russell et al., 2020).

Regarding AD, dietary intervention showed improvement in daily function and quality of life. Notably, the Mediterranean diet exhibits a reduced risk of AD, particularly among those with the highest adherence levels (Scarmeas et al., 2006). Similarly, the MIND diet is associated with lower global AD pathology and decreased β-amyloid load. The higher consumption of green leafy vegetables within the diet correlated with reduced AD pathology (Agarwal et al., 2023). In the case of the ketogenic diet, it has demonstrated cognitive improvements and enhanced quality of life in AD patients across various stages of the disease (Tabaie et al., 2022). Physical activity has been shown to, at least temporarily, enhance certain cognitive functions in individuals with AD, specifically attention, executive functions, and language (Coelho et al., 2009; Vreugdenhil et al., 2012).

Similar positive results have been found concerning PD. A study showed that adhering to the Mediterranean diet led to notable cognitive improvements in PD patients, particularly in executive function, language, attention, concentration, and active memory (Paknahad et al., 2020). Similarly, the MIND diet, was associated with a reduced risk of parkinsonism and a slower progression of parkinsonism in older adults (Agarwal et al., 2018). Finally, the ketogenic diet also led to improvements in motor and non-motor symptoms, particularly in cognitive function and overall well-being (Phillips et al., 2018). Moreover, moderate-intensity physical activity was correlated with enhanced global cognition, visuospatial perception, memory, and executive function. In resting-state, the brainstem, hippocampus, and areas within the frontal, cingulate, and parietal cortices, indicate increased connectivity across the brain (Donahue et al., 2022).

Interestingly, it has also been shown that exercise training upregulates SIRT1 to attenuate inflammation and metabolic dysfunction (Liu et al., 2019). In fact, through the use of a SIRT1 synthetic activator, there was increased fat oxidation, mimicking low energy levels, protecting against diet-induced metabolic disorders (Feige et al., 2008). More recently, Heyward et al. (2016) have demonstrated that in obese mice, upon the supplementation with a SIRT1 activator, resveratrol, hippocampus-dependent memory was preserved, thus suggesting another mechanism by which obesity can affect cognition.

While many studies support the role of diet and physical activity changes in reducing both the risk of developing these diseases or alleviating respective severities, there is still a long path ahead. The use of several different diets and physical activities across studies makes it difficult to find the diet or physical activity with the best outcomes for each disease. Once the best diet plan for each disease is determined, it’s worth considering making small personalized changes based on each person’s metabolic profiles. This approach would probably promote better outcomes.

Starting with the development of more consistent and reproducible protocols or strategies for each disease, as well as larger longitudinal studies should be addressed in future studies, along with the assessment of further roles of leptin, adiponectin and SIRT1.

## 7. Where we stand and where must we go?

Above, in Table 1, there is an overview of the major findings associated with obesity in MS, AD, and PD. These significant discoveries offer valuable insights into the complex relationships between these neurological diseases and obesity-related factors.

**TABLE 1:** Major mechanistic and molecular findings in multiple sclerosis, Alzheimer’s disease, and Parkinson’s disease.

Disease	Major findings	References
Multiple sclerosis	Obesity promotes blood-brain barrier disruption, facilitating the infiltration of immune cells	Davanzo et al., 2023
	Leptin and adiponectin imbalances impact immune responses and neuroinflammation	Matarese et al., 2005; Frisullo et al., 2007; Piccio et al., 2013; Marrodan et al., 2021
	Oligodendrocyte loss is promoted through oxidative stress produced by obesity	Langley et al., 2020
	Gut microbiota dysbiosis contributes to disease severity and progression	Shahi et al., 2022
Alzheimer’s disease	Obesity-associated insulin resistance, oxidative stress, and inflammation accelerate Aβ peptide production and neuroinflammation	Zhao et al., 2008; Di Carlo et al., 2012; Nuzzo et al., 2015; Ng and Chan, 2017
	Leptin resistance impact Aβ peptide accumulation and synaptic dysfunction	Bonda et al., 2014
Parkinson’s disease	High-fat diet-induced neuroinflammation, astrogliosis and mitochondrial dysfunction contribute to dopaminergic neuron degeneration	Kao et al., 2020
	Tyrosine hydroxylase is found decreased due to obesity-induced insulin resistance, inflammation, oxidative stress, and activation of astroglia and microglia	Bittencourt et al., 2022

This table presents major mechanistic and molecular findings in multiple sclerosis, Alzheimer’s disease, and Parkinson’s disease in the context of obesity-related factors.

**FIGURE 2:**
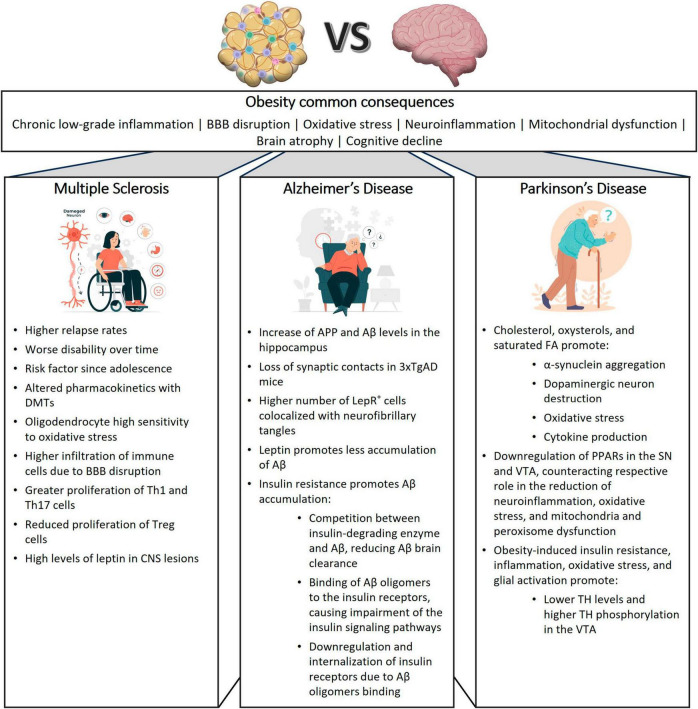
Influence of obesity on neurodegenerative diseases. This figure illustrates the shared and distinctive effects of obesity on multiple sclerosis (MS), Alzheimer’s disease (AD), and Parkinson’s disease (PD), providing insights into the common pathways and disease-specific mechanisms contributing to neurodegeneration in these conditions. 3xTgAD, Triple-transgenic model of Alzheimer’s disease; Aβ, Amyloid-beta; APP, amyloid-beta precursor protein; BBB, blood brain barrier; DMTs, disease modifying treatments; CNS, Central Nervous System; FA, fatty acid; LepR, leptin receptor; PPAR, peroxisome proliferator-activated receptor; SN, substantia nigra, TH, tyrosine hydroxylase; VTA, ventral tegmental area.

Moreover, as depicted in Figure 2, despite several mechanisms that are shared between MS, AD and PD, there are several mechanisms that are distinctive between them. So, it will be crucial to integrate all these data and fill the research gaps to define new therapeutic approaches.

A comprehensive overview of the research gaps identified in the context of obesity-related neurological diseases can be found in Table 2. These gaps highlight areas where further investigation and inquiry are needed to deepen our understanding of the impact of obesity on CNS disorders as MS, AD, and PD.

**TABLE 2:** Research gaps in obesity-related effects on neurological diseases.

Multiple sclerosis	Alzheimer’s disease	Parkinson’s disease
What are the mechanisms through which obesity influences relapse rate and brain volume decline?	How does obesity and cholesterol accumulation in lipid rafts impact the production of Aβ peptide?	May obesity be considered a risk factor for emergence and progression in Parkinson’s disease, given the heterogeneity of results?
Must the disease-modifying therapies dosages be adjusted at the beginning of medication considering obesity-related altered pharmacokinetics?	What is the effect of obesity on the progression and severity of Alzheimer’s disease in different stages of the disease?	What are the specific mechanisms through which obesity affects dopaminergic neurons?
Which specific mechanisms does obesity have on the immune system and its potential impact on multiple sclerosis course?		

This table summarizes the research gaps identified in the three neurological diseases described in this review and respective relation with obesity, providing insights for future research directions.

## 8. Conclusion

In recent years, researchers have uncovered several CNS complications derived from obesity. While the exact mechanisms linking obesity to neurodegeneration remain unclear, it is believed that chronic inflammation, insulin resistance, oxidative stress, and BBB disruption are shared features that further exacerbate CNS diseases. Through continued inquiry and collaboration, this field is expected to yield new insights and innovative approaches to address the complex interplay between obesity and neurodegeneration allowing the development of both effective prevention and treatment strategies to tackle MS, AD, and PD progression, focusing on weight loss and healthier diets.

## Author contributions

AN: Writing – original draft. AF: Writing – review and editing. AB: Conceptualization, Supervision, Writing – original draft, Writing – review and editing.

## Footnotes

1 World Health Organization, “Obesity and overweight”, WHO, 2018, https://www.who.int/news-room/fact-sheets/detail/obesity-and-overweight

2 The Multiple Sclerosis International Federation, “Atlas of MS, 3rd Edition”, MSIF, 2020, https://www.atlasofms.org/map/global/epidemiology/number-of-people-with-ms
